# The endogenous capacity to produce proinflammatory mediators by the ex vivo human perfused lung

**DOI:** 10.1186/s40635-020-00343-x

**Published:** 2020-09-21

**Authors:** Aleksandra Leligdowicz, James T. Ross, Nicolas Nesseler, Michael A. Matthay

**Affiliations:** 1 Cardiovascular Research Institute, University of California, San Francisco, San Francisco, CA USA; 2 Interdepartmental Division of Critical Care Medicine, University of Toronto, Toronto, Ontario Canada; 3 Department of Surgery, University of California, San Francisco, San Francisco, CA USA; 4 Department of Anesthesia and Critical Care, Pontchaillou, University Hospital of Rennes, Rennes, France; 5 Univ Rennes, CHU de Rennes, Inra, Inserm, Institut NUMECAN–UMR_A 1341, UMR_S 1241, 35000 Rennes, France; 6 Univ Rennes, CHU Rennes, Inserm, CIC 1414 (Centre d’Investigation Clinique de Rennes), 35000 Rennes, France; 7 Department of Medicine, Division of Pulmonary, Critical Care, Allergy and Sleep Medicine, University of California, San Francisco, San Francisco, CA USA; 8 Departments of Medicine and Anesthesia, Cardiovascular Research Institute, University of California, San Francisco, San Francisco, CA USA

**Keywords:** Biomarkers, Ex vivo-perfused lung, *Streptococcus pneumoniae*, ARDS, Lung transplant

## Abstract

**Background:**

The ex vivo human perfused lung model has enabled optimizing donor lungs for transplantation and delineating mechanisms of lung injury. Perfusate and airspace biomarkers are a proxy of the lung response to experimental conditions. However, there is a lack of studies evaluating biomarker kinetics during perfusion and after exposure to stimuli. In this study, we analyzed the ex vivo-perfused lung response to three key perturbations: exposure to the perfusion circuit, exogenous fresh whole blood, and bacteria.

**Results:**

Ninety-nine lungs rejected for transplantation underwent ex vivo perfusion. One hour after reaching experimental conditions, fresh whole blood was added to the perfusate (*n* = 55). Two hours after reaching target temperature, *Streptococcus pneumoniae* was added to the perfusate (*n* = 42) or to the airspaces (*n* = 17). Perfusate and airspace samples were collected at baseline (once lungs were equilibrated for 1 h, but before blood or bacteria were added) and 4 h later. Interleukin (IL)-6, IL-8, angiopoietin (Ang)-2, and soluble tumor necrosis factor receptor (sTNFR)-1 were quantified. Baseline perfusate and airspace biomarker levels varied significantly, and this was not related to pre-procurement P_a_O_2_:FiO_2_ ratio, cold ischemia time, and baseline alveolar fluid clearance (AFC). After 4 h of ex vivo perfusion, the lung demonstrated a sustained production of proinflammatory mediators. The change in biomarker levels was not influenced by baseline donor lung characteristics (cold ischemia time, baseline AFC) nor was it associated with measures of experimental epithelial (final AFC) or endothelial (percent weight gain) injury. In the presence of exogenous blood, the rise in biomarkers was attenuated. Lungs exposed to intravenous (IV) bacteria relative to control lungs demonstrated a significantly higher rise in perfusate IL-6.

**Conclusions:**

The ex vivo-perfused lung has a marked endogenous capacity to produce inflammatory mediators over the course of short-term perfusion that is not significantly influenced by donor lung characteristics or the presence of exogenous blood, and only minimally affected by the introduction of systemic bacteremia. The lack of association between biomarker change and donor lung cold ischemia time, final alveolar fluid clearance, and experimental percent weight gain suggests that the maintained ability of the human lung to produce biomarkers is not merely a marker of lung epithelial or endothelial injury, but may support the function of the lung as an immune cell reservoir.

## Background

The ex vivo human perfused lung has been used for nearly 70 years to study mechanisms of lung function [1]. The model facilitates characterizing biological mechanisms that may preserve the lung for transplantation as well as response to clinically relevant pathological conditions, such as exposure to endotoxin or bacteria [1–3]. The experimental preparation has improved the available pool of lungs appropriate for transplantation [4–9] and understanding mechanisms that contribute to primary graft dysfunction in lung transplant recipients [10]. It also offers insight into lung physiology in injury [11, 12] and as such, allows for the testing of new therapeutics (mesenchymal stem cells, microvesicles) [13–16].

The controlled conditions of the ex vivo*-*perfused lung make it possible to collect samples from multiple compartments (perfusate, lung tissue, airspaces) and to study the response to experimental intervention. The quantification of biomarkers in these compartments is representative of injury [17–20]. However, whether biomarker levels are associated with a negative outcome is uncertain in the ex vivo*-*perfused lung model because the rise in biomarkers does not always correlate with validated measures of lung function [21]. Furthermore, removing biomarkers thought to induce injury, such as IL-8, with an adsorbent membrane, does not improve lung function during prolonged ex vivo lung perfusion (EVLP) [22].

The uncertainty in the field prompted us to perform a detailed study of several clinically relevant biomarkers that have been studied in clinical samples as well as in ex vivo and in vitro models of lung injury. The four biomarkers include three biomarkers associated with inflammation: interleukin (IL)-6, IL-8, and soluble tumor necrosis factor receptor 1 (sTNFR1) [23–25], as well as one biomarker associated with endothelial activation: angiopoietin-2 (Ang-2) [25, 26].

Additionally, the human EVLP model has been shown to lack a response to the airspace administration of bacterial components (lipopolysaccharide, LPS) without the addition of exogenous blood [14]. Therefore, in many instances where the ex vivo human perfused lung model is used for studying the response to injurious stimuli, exogenous fresh human whole blood has been added to the perfusate [1, 15]. However, the influence of the addition fresh whole blood to the ex vivo human perfused lung on pro-inflammatory biomarker levels in the perfusate and in the airspaces has not been studied in detail.

The goal of our study was to address four important poorly understood concepts. First, do donor lung characteristics (P_a_O_2_:FiO_2_ ratio, cold ischemia time, baseline alveolar fluid clearance [3]) influence baseline biomarker levels or biomarker kinetics in the perfusate and airspaces? Second, what is the impact of exogenous fresh whole blood on biomarker kinetics in the perfusate and in the airspaces during EVLP? Third, what is the impact of exposure to bacteria (intravenous (IV) or airspace infection with *Streptococcus pneumoniae*) on perfusate and airspace biomarker kinetics relative to uninfected control lungs? Fourth, do donor lung characteristics influence relationships between biomarker kinetics in the presence of exogenous blood or after exposure to bacteria?

## Results

### Donor lung characteristics

A total of 99 single human lungs from 99 donors were studied under the following six experimental conditions: (1) control lungs with exogenous blood (*n* = 22), (2) intravenous infection with *S. pneumoniae* with exogenous blood (*n* = 19), (3) airspace infection with *S. pneumoniae* with exogenous blood (*n* = 14), (4) control lungs without exogenous blood (*n* = 18), (5) intravenous infection with *S. pneumoniae* without exogenous blood (*n* = 23), (6) airspace infection with *S. pneumoniae* without blood (*n* = 3). The baseline donor characteristics among the six experimental conditions were well balanced (Table 1). There were no statistically significant differences in donor age, organ cold ischemia time, PaO_2_:FiO_2_ ratio, and baseline alveolar fluid clearance (AFC). Among the lungs perfused without exogenous blood, a greater proportion of donors of control lungs relative to donors of lungs exposed to intravenous bacteria received pre-procurement antibiotics (89% vs 52%, *p* = 0.01). This imbalance in pre-procurement antibiotics was greater in control lungs and therefore would not be expected to affect bacterial proliferation.

**Table 1:** Characteristics of the 99 donor lungs stratified by 6 experimental conditions.

	**With blood** ***n*=55**			**Without Blood** ***n*=44**			***P*-value**
	**Control lungs** ***n*=22**	**IV bacteria** ***n*=19**	**Airspace bacteria** ***n*=14**	**Control lungs** ***n*=18**	**IV bacteria** ***n*=23**	**Airspace bacteria** ***n*=3**	
**Donor age** ^b^	44 (30, 55)	45 (32, 54)	45.5 (30, 55)	40 (38, 46)	53 (32, 64)	59 (55, 69)	0.4
**Donor sex**	15 (68%)	13 (68%)	9 (64%)	11 (61%)	16 (70%)	3 (100%)	0.9
**Donor weight (kg)** ^b^	88.5 (83.1, 104.3)	86.9 (72.7, 96)	83.9 (66.5, 112)	89.7 (75, 104)	83 (73.4, 96)	108 (85, 171)	0.5
**Donor height (cm)** ^a^	172.5 (±14.5)	174.4 (±7)	172.1 (±8.8)	173.7 (±10.8)	169.9 (±10.6)	179.3 (±4)	0.5
**Cold ischemia Time (h)** ^a^	20 (±13.5)	23.7 (±13.3)	19.3 (±14)	24.6 (±10.6)	19.7 (±10)	28 (±8.5)	0.6
**Pre-procurement antibiotics**	13 (59%)	13 (68%)	13 (93%)	16 (89%)	12 (52%)	2 (67%)	0.05
**NDD**	19 (86%)	18 (95%)	13 (93%)	17 (94%)	20 (87%)	3 (100%)	0.9
**Trauma**	6 (27%)	5 (26%)	4 (29%)	5 (28%)	8 (35%)	2 (67%)	0.8
**CPR**	14 (64%)	10 (53%)	10 (71%)	9 (50%)	12 (52%)	1 (33%)	0.7
**PaO**_**2**_**:FiO**_**2**_ **ratio** ^a^	224 (±74)	268 (±103)	237 (±136)	251 (±111)	242 (±100)	251 (±104)	0.7
**Normal baseline AFC**	17 (77%)	15 (79%)	11 (79%)	16 (89%)	20 (87%)	2 (67%)	0.9
**AFC at baseline** ^b^	33.4 (9.5, 45.5)	27.3 (9.2, 40)	18.4 (15.4, 32.2)	28.8 (15, 33.3)	28 (18.2, 38.9)	20.6 (4.3, 49.1)	0.9
**AFC at 5h** ^b^	14 (9.5, 36.2)	18.7 (9.5, 29.7)	2.7 (0, 16.9)	22.7 (14.8, 32)	15.2 (13.3, 32.4)	5 (0, 10)	0.02
**Start lung weight (g)** ^b^	402 (330, 532)	383 (351, 438)	372 (294, 396)	365 (297, 406)	360 (328, 414)	555 (373, 562)	0.2
**End lung weight (g)** ^b^	631 (564, 958)	784 (720, 1232)	767 (603, 891)	722 (630, 774)	779 (696, 987)	1328 (1312, 1344)	0.1
**% weight gain** ^b^	62 (44, 99)	115 (69, 186)	98 (83, 154)	99 (80, 128)	101 (82, 134)	138 (133, 142)	0.04

*Abbreviations: *NDD* Neurological declaration of death, *CPR* Cardiopulmonary resuscitation, *AFC* Alveolar fluid clearance

*Continuous data presented as means ± standard deviation (^a^) or medians with interquartile range (^b^)

*Group comparisons of continuous variables made using Kruskal–Wallis test

### Baseline biomarker variability and significant biomarker rise after 4 h of EVLP

**Fig. 1:**
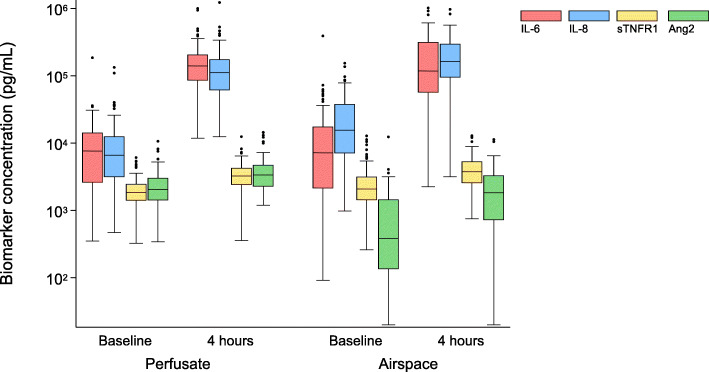
Perfusate and airspace biomarker concentrations at baseline and after 4 h of ex vivo perfusion. Biomarker concentrations are presented on a Log_10_ scale. The capacity to produce the four inflammatory cytokines at the 4 hour relative to the 0-h time point in both the perfusate and in the airspace compartment was significant at *p* < 0.0001 for all presented data

Because the first sample was collected before the addition of experimental conditions (exogenous fresh whole blood, intravenous or airspace infection with *S. pneumoniae*), we were able to study baseline biomarker levels in the perfusate (*n* = 97) and in the airspace (*n* = 88). Baseline (time 0 h) perfusate and airspace samples collected 1 h after EVLP equilibration had a wide distribution in biomarker concentrations (Fig. 1).

Perfusate and airspace samples collected at baseline and after 4 h of EVLP were used to study biomarker kinetics. The ex vivo human perfused lung demonstrated a maintained capacity to produce inflammatory cytokines at 4 h relative to the 0-h time point in the perfusate and in the airspaces (Fig. 1) at a significance level of *p* < 0.0001. The substantial fold change in perfusate and airspace biomarkers is shown in sFigure 1.

A significant rise in the perfusate (sTable 1) and airspace (sTable 2) biomarkers was present in all six experimental conditions. This was especially true for IL**-**6 and IL**-**8 (sFigure 2A**-**B and sFigure 3A**-**B), with IL**-**6 levels increasing more than 200-fold (sFigure 1) up to a concentration of 1 μg/ml. The increase in perfusate and airspace sTNFR1 and Ang-2 was lower (sFigure 2C-D and sFigure 3C-D).

### Donor characteristics and indicators of experimental lung injury are not associated with baseline biomarker levels and biomarker kinetics

**Fig. 2:**
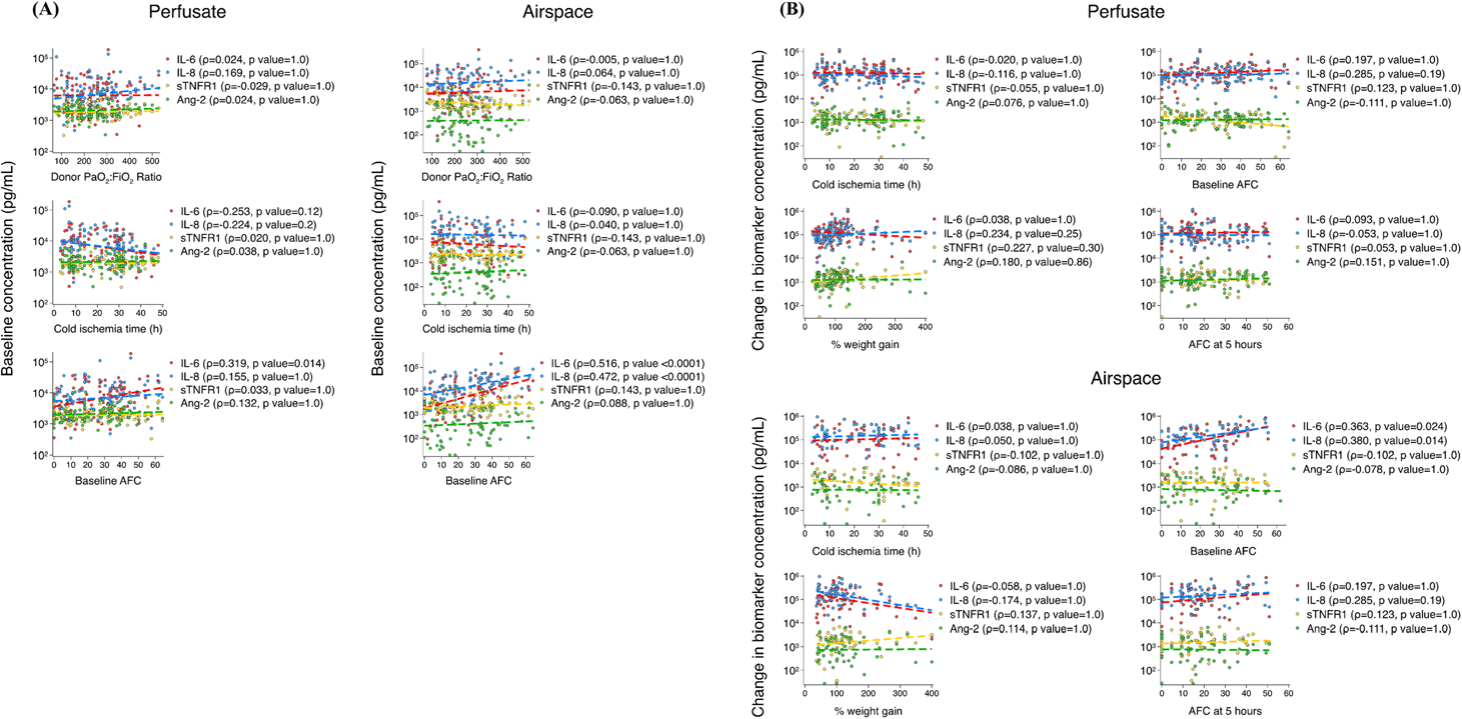
Perfusate and airspace biomarkers at baseline and change after perfusion relative to lung characteristics. Associations between (**a**) baseline perfusate (*n* = 97) and airspace (*n* = 88) biomarker levels and donor lung characteristics, and (**b**) change in perfusate (*n* = 95) and airspace (*n* = 65) biomarker concentrations after 4 h of ex vivo lung perfusion and donor lung characteristics and indicators of lung epithelial and endothelial injuries at the end of the experiment. *p* values are presented after a Bonferroni correction for multiple testing

Baseline biomarker levels were tested for their association with pre-procurement P_a_O_2_:FiO_2_ ratio, cold ischemia time, and baseline AFC. Neither perfusate nor airspace baseline biomarker concentrations were associated with these donor lung characteristics (Fig. 2a). The only exception was for airspace IL-6 and IL-8 levels, whereby a higher baseline AFC was associated with a higher baseline biomarker concentration (*p* < 0.0001).

In spite of a substantial rise in perfusate and airspace IL-6 and IL-8, as well as to a lesser extent in sTNFR1 and Ang-2, there was no association between the change in any of the perfusate biomarker levels and indicators of lung injury at the end of the experiment (percent weight gain or final AFC) or baseline donor lung characteristics (Fig. 2b).

### Exogenous blood attenuates perfusate IL-6 increase in lungs exposed to IV *S. pneumoniae*

**Fig. 3:**
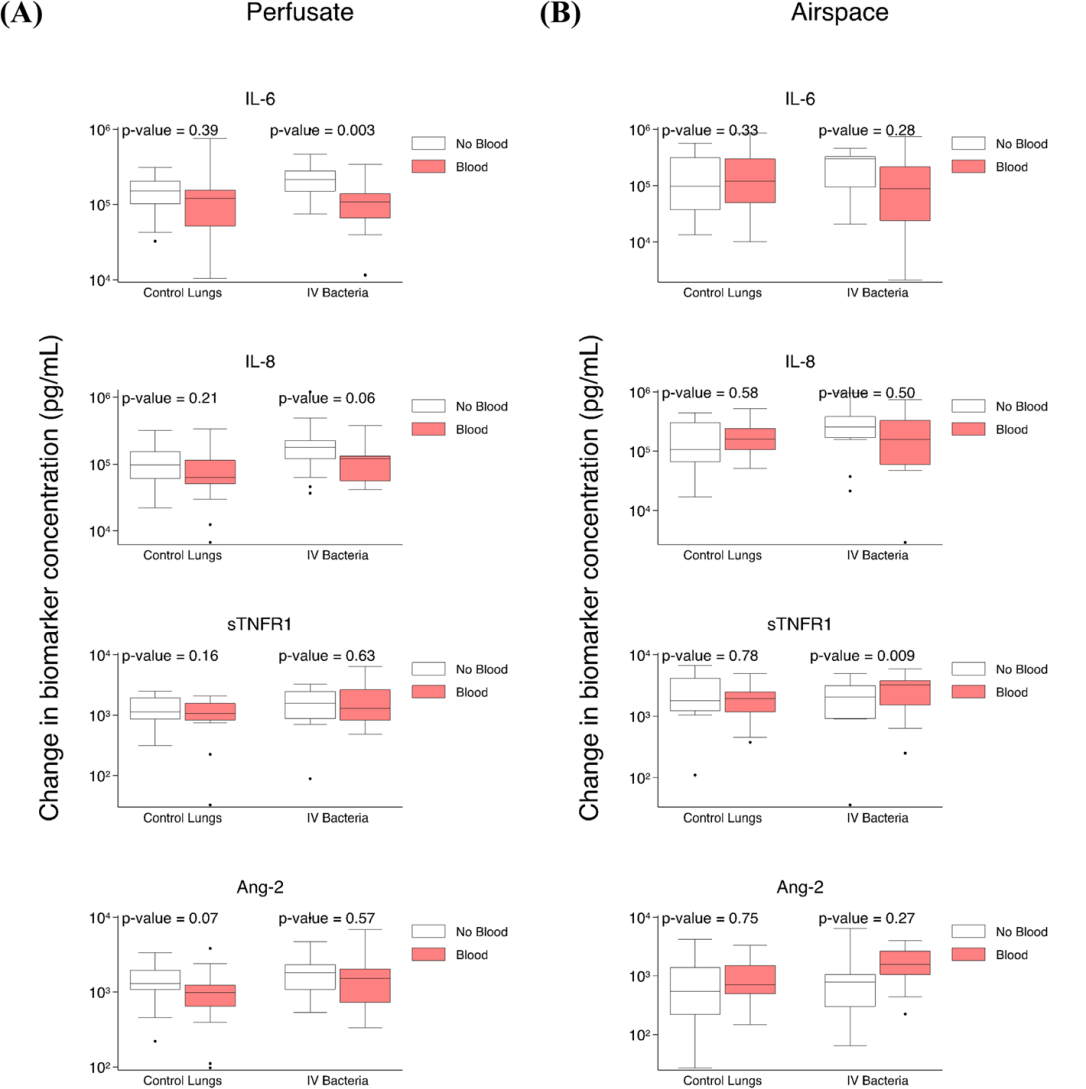
Impact of exogenous blood on biomarker change after 4 h of ex vivo lung perfusion. **a** Comparison of change in perfusate biomarker levels after 4 h of ex vivo perfusion in control lungs in the presence (*n* = 21) or in the absence (*n* = 17) of exogenous blood, and in IV *S. pneumoniae* exposed lungs in the presence (*n* = 19) or in the absence (*n* = 22) of exogenous blood. **b** Comparison of change in airspace biomarker levels after 4 h of ex vivo perfusion in control lungs in the presence (*n* = 16) or in the absence (*n* = 13) of exogenous blood, and in IV *S. pneumoniae*-exposed lungs in the presence (*n* = 13) or in the absence (*n* = 14) of exogenous blood

The differences between perfusate and airspace biomarker kinetics after 4 h of ex vivo perfusion in the presence relative to the absence of fresh whole blood was compared in control lungs and in lungs exposed to IV *S. pneumoniae* (sTable 3). In control lungs, there were no statistically significant differences in the change in perfusate (Fig. 3a) or airspace (Fig. 3b) biomarkers after 4 hours of perfusion in lungs perfused with compared to without exogenous blood. In lungs exposed to IV *S. pneumoniae*, there was a significant difference in the increase in perfusate IL-6 (Fig. 3a) and airspace sTNFR1 (Fig. 3b) in lungs perfused with relative to without exogenous blood. In the absence of blood, perfusate IL-6 increase was 133,885 pg/ml higher relative to lungs perfused with blood (*p* = 0.003). In the presence of blood, airspace sTNFR1 increase was 1,793 pg/ml higher relative to lungs without blood (*p* = 0.009).

Adjustment for potential confounders (donor lung cold ischemia time and baseline AFC) in control lungs compared with lungs perfused with or without exogenous blood had minimal influence on perfusate and airspace biomarker kinetics (sTable 4). None of the significant biomarker kinetics discussed above were changed after adjustment.

### IV *S. pneumoniae* contributes to an increase in perfusate IL-6 in the absence of exogenous blood

To address the question of whether there would be a difference in the increase in lung endogenous biomarker production after *S. pneumonia* exposure relative to control lungs, we studied (1) control lungs relative to lungs exposed to IV *S. pneumoniae* with exogenous blood, (2) control lungs relative to lungs exposed to IV *S. pneumoniae* without blood, and (3) control lungs relative to lungs exposed to airspace *S. pneumoniae* with exogenous blood (sTable 5).

**Fig. 4:**
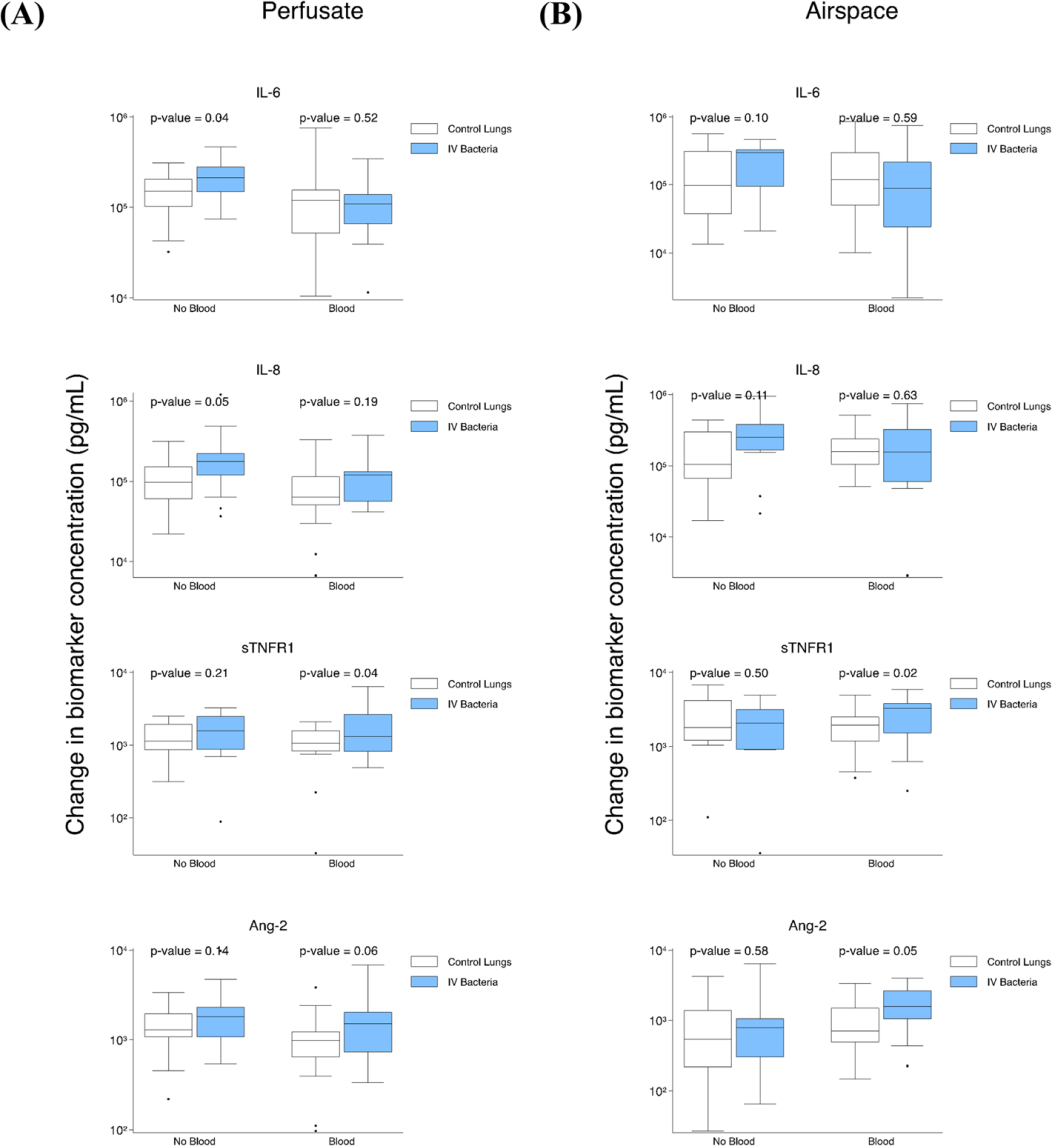
Impact of IV *S. pneumoniae* on biomarker change levels after ex vivo lung perfusion. **a** Comparison of change in perfusate biomarker levels after 4 h of ex vivo perfusion in control lungs (*n* = 17) relative to models with IV *S. pneumoniae* (*n* = 22) without blood and in control lungs (*n* = 21) relative to models with IV *S. pneumoniae* (*n* = 19) with blood. **b** Comparison of change in airspace biomarker levels after 4 h of ex vivo perfusion in control lungs (*n* = 13) relative to models with IV *S. pneumoniae* (*n* = 14) without blood and in control lungs (*n* = 16) relative to IV *S. pneumoniae* (*n* = 13) with blood

Lungs exposed to IV *S. pneumoniae* perfused without exogenous blood relative to control lungs had a significantly greater increase in perfusate IL-6 (higher by 92,741 pg/ml, *p* = 0.04) and perfusate IL-8 (higher by 103,915 pg/ml, *p* = 0.05) (Fig. 4a). There were no differences in airspace biomarker levels (Fig. 4b). When exogenous blood was present in the model, lungs exposed to IV *S. pneumoniae* relative to control lungs had a greater increase in perfusate sTNFR1 (higher by 967 pg/ml, *p* = 0.04) (Fig. 4a), airspace sTNFR1 (higher by 1,492 pg/ml, *p* = 0.02), and airspace Ang-2 (higher by 823 pg/ml, *p* = 0.05) (Fig. 4b). In lungs exposed to airspace *S. pneumoniae* relative to control lungs, there was no significant difference between perfusate or airspace biomarker kinetics (sTable 5).

Adjustment for donor lung characteristics (cold ischemia time, baseline AFC, and pre-procurement antibiotics) in control lungs compared with lungs exposed to IV (sTable 6) and airspace (sTable 7) *S. pneumoniae* had minimal impact on perfusate and airspace biomarker kinetics. Furthermore, none of the significant biomarker kinetics were influenced by these adjustment variables.

## Discussion

In the USA, lungs of approximately 75% eligible donors are ineligible for transplantation [27]. These lungs are an invaluable resource to study lung function and organ preservation to increase eligibility for transplantation. In this study, explanted human lungs rejected for transplantation underwent ex vivo lung perfusion and a detailed characterization of perfusate and airspace biomarker kinetics was performed under 6 experimental conditions.

During 4 h of EVLP, we found that the lung has a remarkable capacity to produce proteins associated with immune (IL-6, IL-8, sTNFR1) and endothelial (Ang-2) responses. The baseline level and the change in concentration of these mediators after 4 h of ex vivo perfusion was unrelated to the duration of cold ischemia time and parameters associated with deranged lung epithelial (alveolar fluid clearance) or endothelial (percent weight gain) function [1]. The lack of association between change in biomarker levels and the final AFC and percent weight gain suggests that the presence of very high levels of proinflammatory mediators does not result in deterioration of epithelial or endothelial barrier function. The weak but significant association between baseline AFC and the increase in airspace IL-6 and IL-8 levels, may suggest that lungs with higher baseline AFC have a higher rise in airspace concentrations of these two inflammatory markers. The addition of fresh whole blood attenuated the increase in perfusate IL-6 in lungs exposed to intravenous bacteria. The addition of a lethal dose of Gram-positive bacteria (*S. pneumoniae*) did not significantly change perfusate or airspace biomarker kinetics, with the exception of perfusate IL-6 in the absence of exogenous blood.

A similar high rise in some biomarkers quantified in this study has been demonstrated in prior reports of EVLP [20, 28]. IL-6 and IL-8 are inflammatory cytokines traditionally synonymous with organ injury and poor outcome. In the EVLP model, several studies of cross-sectional cytokine levels suggest that donor lung levels of these biomarkers are inversely associated with graft function in the transplant recipient [17–20]. Interestingly, cytokines in the EVLP perfusate of successfully transplanted lungs also reach extremely high levels (as much as a 30–100-fold increase in IL-6 and IL-8) without evidence of primary graft dysfunction (PGD) [20, 21]. If IL-6 and IL-8 are always injurious, why do not more transplant recipients develop PGD?

High levels of IL-6, IL-8, sTNFR1, and Ang-2 in plasma samples of patients with the acute respiratory distress syndrome (ARDS) are well-known for their association with poor outcomes [29, 30]. However, plasma biomarker levels are substantially lower than those reported in EVLP perfusate. This discrepancy between the extremely high levels of inflammatory cytokines in the EVLP perfusate and the lack of injury in every transplant recipient as well as the lack of association with experimental measurements of injury in our study (percent weight gain, final AFC) suggest that these cytokines have an additional biologic significance [31], especially in the EVLP model. Future studies may provide insight into the biologic relevance of the elevated biomarker levels in the EVLP model, including their potential role in immune cell recruitment, enhancement of phagocytosis, stimulation of tissue turnover, or eradication of pathogens.

The source of biomarker production and the mechanism of accumulation are uncertain. The EVLP model lacks mechanisms associated with clearance, specifically the liver [32], the kidneys [33], and components of the vascular compartment, the absence of which may explain the striking difference between biomarker concentrations detectable in plasma relative to the perfusate and airspaces of the ex vivo perfused lung. In our experiments in which only 100 ml of fresh whole blood was added to 2 l of EVLP perfusate (representative of a hematocrit of approximately 2%), there appeared to be a trend toward a dampening effect on the increase in perfusate IL-6 and IL-8 levels in control lungs as well as in lungs exposed to IV *S. pneumoniae.* This suggests that protective factors are present in blood that either reduce the production of these cytokines, that increase their enzymatic clearance, or that facilitate their sequestration [34]. Future investigation of whole blood components that may be responsible for the decrease in inflammatory biomarker levels may be relevant to transplantation as several studies support the notion that high IL-8 in perfusate is related to an increased incidence of PGD3 in the recipient [17–19].

A potential source of cytokines may be cell necrosis and apoptosis due to ischemia-reperfusion injury [35]. However, neither the baseline nor the change in biomarkers after 4 h of EVLP was associated with cold ischemia time, suggesting that presumed cell necrosis and apoptosis cannot on their own explain the high abundance of cytokines in our model. In fact, prolonged hypothermia without reperfusion did not increase pneumocyte apoptosis in a rat lung transplant model [36]. Also, there is evidence from studying tissue biomarker levels in the human EVLP model that inflammatory cytokine levels do not significantly differ after extended cold ischemia time [7].

Of note, not all biomarker levels increased to the same extent during EVLP. The small change in Ang-2 levels during the 4 h of ex vivo perfusion relative to inflammatory biomarker levels is puzzling. This protein is produced by the endothelium and is stored in Weibel Palade bodies [26], where it has a long half-life (over 18 h) and can be secreted within minutes of stimulation [37]. However, despite lung injury due to ischemia-reperfusion and addition of lethal doses of *S. pneumoniae*, the magnitude of Ang-2 change was minimal compared with the change in IL-6 and IL-8.

The observation that starting AFC is only significantly associated with baseline IL-6 and IL-8 levels in the airspace compartment is intriguing. It is plausible that when the lung epithelial function is intact and alveolar fluid clearance is preserved [38], IL-6 and IL-8 may concentrate in the airspaces. It also implies that when interpreting levels of these cytokines in the airspace compartment of the EVLP model, it is important to measure and account for lung alveolar fluid clearance.

This study has some limitations. First, only two time points were studied during the course of perfusion, and as such, it is possible we missed important time point biomarker production trends, particularly in experimental conditions in the presence of bacteria. It is also possible that the perfusion was not long enough to appreciate differences between the experimental conditions included in this study. Secondly, we studied only four soluble proteins, and to detect relevant differences between experimental conditions, other proteins may need to be studied. It is also plausible that other metrics, such as RNA expression, microvesicle or lipid production, could provide insight into biological differences between the experimental conditions. Third, our experiments were performed with a single bacterial pathogen, and as such, the data may not be extrapolated to infection with other pathogens. Lastly, the effect of exogenous whole blood on biomarker kinetics in lungs exposed to airspace *S. pneumoniae* could not be studied as there were only 3 lungs in this subgroup. Therefore, the previous observation that the addition of whole blood leads to a sharp increase in airspace biomarkers (IL-1β, TNF⍺, and IL-8) when a bacterial component (LPS) is instilled into one of the lung lobes [14] could not be ascertained.

## Conclusions

Overall, the ex vivo-perfused lung has a remarkable capacity to generate high levels of inflammatory proteins during 4 h of perfusion. This was true in all experimental conditions, including control lungs, suggesting that future EVLP models assessing effect of injury or novel therapies should incorporate appropriate control lungs in the study design. The substantial increase in biomarker levels between the two time points suggests that future studies of cross-sectional and longitudinal biomarker analysis should ensure strict adherence to predefined timing of sample collection. The addition of blood does not increase biomarker levels, while the addition of live bacteria results in a higher rise in only IL-6 in lungs perfused without exogenous blood. The remarkable lack of association between baseline proinflammatory biomarker levels as well as their increase over time and donor lung characteristics (cold ischemia time, starting AFC) and experimental outcomes (alveolar fluid clearance 5 h after perfusion and the percent weight gain) suggests that the maintained ability to produce biomarkers is not merely a marker of lung epithelial or endothelial injury and may instead support the lung’s role as an immune reservoir.

## Materials and methods

### Ex vivo human perfused lung

Donor lungs rejected for transplantation were received from Donor Network West. Lungs were rejected for various reasons, including a mismatch in sex, race, size, or geography between the donor and available recipients, donor age or smoking history, radiographic or bronchoscopic findings suggestive of atelectasis, edema and/or infection, or other elements of the donor history or clinical course [39, 40]. The right or left lung was selected for EVLP based on gross appearance, as previously described [1]. Briefly, the main bronchus was intubated with an endotracheal tube and the pulmonary artery was cannulated. The lung was perfused with 2 l of acellular DME-H21 media with 5% bovine serum albumin (BSA) and warmed to 37 °C. Subsequently, 8 cm H_2_O of continuous positive airway pressure was applied using room air. Lung weight was obtained at the start (baseline) and at the end of each experiment. One hour after experimental conditions were reached, 100 ml of exogenous fresh whole blood was added to the perfusate of some of the lungs (sFigure 4). University of California, San Francisco (UCSF) Institutional Review Board (IRB) approval was obtained for the collection of blood from healthy volunteers (laboratory members).

### Quantification of lung function: alveolar fluid clearance

Alveolar fluid clearance (AFC) was determined once the ex vivo*-*perfused lung was equilibrated on the circuit by introducing 100 ml of normal saline with 5% BSA into the distal airspaces. Samples for measurement of total protein by refractometry were collected at 5 and 35 min via a catheter inserted into the endobronchial tube. AFC was calculated using the formula: AFC (%/h) = 2(1 − *C*_*i*_/*C*_*f*_), where *C*_*i*_ is the 5-min sample protein concentration and *C*_*f*_ is the 35-min sample protein concentration [3, 41]. The AFC for all experiments was calculated at the start of the experiment (baseline) and at 5 h (sFigure 4).

### Infection with *Streptococcus pneumoniae*

*S. pneumoniae* serotype 19F (49619; ATCC, Manassas, VA) was grown in brain-heart broth (Becton-Dickinson, Sparks MD), and 10^10^ bacteria was resuspended in phosphate-buffered saline for administration according to a weight-based adjustment of a severe pneumonia murine model [42, 43]. Two hours after reaching target temperature, bacteria were added intravenously into the perfusate (non-pulmonary sepsis model) or into the airspaces (pneumonia model, sFigure 4).

### Biomarker quantification

Perfusate and airspace samples were prospectively collected for biomarker quantification at two time points. The first sample was collected after the lungs were equilibrated on the circuit for 1 h (immediately prior to the addition of exogenous whole blood and 1 h prior to the introduction of *S. pneumoniae* in selected experiments; sample referred to as time 0 h). The second sample was collected 4 h later (sample referred to as time 4 h, sFigure 4). Airspace fluid samples were not available for 11 lungs at the 0-h timepoint and 24 lungs at the 4-h time. This was due to mucus production or very effective AFC which prevented airspace fluid collection. For lungs perfused with exogenous fresh whole blood, this corresponded to a 4-h exposure to blood. For lungs exposed to *S. pneumoniae*, this corresponded to a 3-h exposure to bacteria. All samples were cryopreserved at − 80 °C prior to protein quantification using the Simple Plex^TM^ Ella multiplex microfluidic platform (Protein Simple, CA, USA). A 4-plex custom panel was used to quantify interleukin (IL)-6, IL-8, angiopoietin (Ang)-2, and soluble tumor necrosis factor receptor (sTNFR)-1. Low-abundance proteins (Ang-2) were tested at a 1:10 dilution, and high-abundance proteins (IL-6, IL-8, sTNFR-1) were tested at a 1:1000 dilution. The same dilutions were used for perfusate and airspace samples. Assays were performed according to manufacturer’s protocol, as described previously [44]. Raw data were analyzed using the SimplePlex Explorer software.

### Statistical analysis

Relationships between continuous variables were performed using Pearson (normally distributed data) or Spearman (skewed data) correlation, with a Bonferroni correction for multiple testing. Comparison of baseline continuous variables among experimental groups were analyzed using the Kruskal–Wallis test and dichotomous variables were compared using the chi squared test. Comparisons of biomarker kinetics among experimental conditions were made using generalized estimating equation models (GEE) using robust standard errors [45]. Lungs lacking a sample at the second time point (t4h) were excluded from analysis. The analyses included (1) impact of exogenous whole fresh blood on change in perfusate and airspace biomarker levels in lung subgroups stratified by exposure to bacteria (control/no bacteria, intravenous bacteria), (2) impact of addition of intravenous *S. pneumoniae* (non-pulmonary sepsis model) on change in perfusate and airspace biomarker levels in lung subgroups stratified by addition of exogenous whole fresh blood, and (3) impact of addition of airspace *S. pneumoniae* (pneumonia model) on change in perfusate and airspace biomarker levels in lungs perfused with exogenous whole fresh blood. Too few lungs exposed to airspace *S. pneumoniae* were perfused without exogenous blood (*n* = 3) to test the impact of airspace bacteria on biomarker kinetics in this experimental subgroup. Interaction terms for cold ischemia time, baseline lung function (the surrogate of which was baseline AFC), lung response at end of experiment (AFC at 5 h, percent weight gain) were included individually in the above GEE models to test for the effect of these potential confounders on perfusate and airspace biomarker kinetics. In models assessing the impact of bacteria on biomarker kinetics, an interaction term for the administration of pre-procurement antibiotics was included. Statistical analyses and data presentation were performed using STATA v14.1 (StataCorp 2015).

## Supplementary information

**Additional file 1.** Supplementary Figures and Tables.

## Data Availability

The datasets used and/or analyzed during the current study are available from the corresponding author on reasonable request.

## Abbreviations

IL-6: Interleukin-6
IL-8: Interleukin-8
Ang-2: Angiopoietin-2
sTNFR1: Soluble tumor necrosis factor receptor-1
AFC: Alveolar fluid clearance
IV: Intravenous
EVLP: Ex vivo lung perfusion
*S. pneumoniae*: *Streptococcus pneumoniae*
LPS: Lipopolysaccharide
