# Supplementary material for: The endogenous capacity to produce proinflammatory mediators by the ex vivo human perfused lung
Source: Intensive Care Med Exp. 2020 Sep 21;8:56. doi: 10.1186/s40635-020-00343-x (PMC7505905; doi:10.1186/s40635-020-00343-x)
Supplement: Supplementary file 1 — Additional file 1. Supplementary Figures and Tables. [file 40635_2020_343_MOESM1_ESM.docx]

**Supplementary Material**

**Table of Contents**

[Supplementary Figures 2](#_Toc47449047)

[sFigure 1: Fold change in perfusate (n=95) and airspace (n=68) biomarker concentrations after 4 hours of *ex vivo* lung perfusion in all experimental conditions combined. Biomarker fold change is presented on a Log_10_ scale. 2](#_Toc47449048)

[sFigure 2: Perfusate biomarkers at 0 and 4 hours stratified by experimental conditions. 3](#_Toc47449049)

[**A.** IL-6 3](#_Toc47449050)

[**B.** IL-8 3](#_Toc47449051)

[**C.** sTNFR1 4](#_Toc47449052)

[**D.** Ang-2 4](#_Toc47449053)

[sFigure 3: Airspace biomarkers at 0 and 4 hours stratified by experimental conditions. 5](#_Toc47449054)

[**A.** IL-6 5](#_Toc47449055)

[**B.** IL-8 5](#_Toc47449056)

[**C.** sTNFR1 6](#_Toc47449057)

[**D.** Ang-2 6](#_Toc47449058)

[sFigure 4: Experimental timeline. 7](#_Toc47449059)

[Supplementary Tables 8](#_Toc47449060)

[sTable 1: Perfusate biomarker kinetics stratified by experimental conditions. 8](#_Toc47449061)

[sTable 2: Airspace biomarker kinetics stratified by experimental conditions. 9](#_Toc47449062)

[sTable 3: Impact of exogenous fresh whole blood on perfusate and airspace biomarker change during 4 hours of *ex vivo* perfusion in control lungs and in lungs exposed to IV *S. pneumoniae*. 10](#_Toc47449063)

[sTable 4: Impact of cold ischemia time and baseline AFC on relationship of perfusate and airspace biomarker change in lungs perfused with compared to without blood in control and IV *S. pneumoniae* exposed lungs. 11](#_Toc47449064)

[sTable 5: Impact of bacteria on perfusate and airspace biomarker change during 4 hours of *ex vivo* perfusion in control lungs relative to lungs exposed to IV *S. pneumoniae* with blood, IV *S. pneumoniae* without blood, or airspace *S. pneumoniae* with blood. 12](#_Toc47449065)

[sTable 6: Impact of cold ischemia time, baseline AFC, and pre-procurement antibiotics on relationship of perfusate and airspace biomarker change in control compared to IV *S. pneumoniae* exposed lungs perfused with and without blood. 13](#_Toc47449066)

[sTable 7: Impact of cold ischemia time, baseline alveolar fluid clearance, and pre-procurement antibiotics on relationship of perfusate and airspace biomarker change in control compared to airspace *S. pneumoniae* exposed lungs perfused with blood. 14](#_Toc47449067)

# Supplementary Figures

## sFigure 1: Fold change in perfusate (n=95) and airspace (n=68) biomarker concentrations after 4 hours of *ex vivo* lung perfusion in all experimental conditions combined. Biomarker fold change is presented on a Log_10_ scale.

## sFigure 2: Perfusate biomarkers at 0 and 4 hours stratified by experimental conditions.

### IL-6

### IL-8

### sTNFR1

### Ang-2

## sFigure 3: Airspace biomarkers at 0 and 4 hours stratified by experimental conditions.

### IL-6

### IL-8

### sTNFR1

### Ang-2

## sFigure 4: Experimental timeline.

# Supplementary Tables

## sTable 1: Perfusate biomarker kinetics stratified by experimental conditions.

|  |  |  | **With blood**  **n=53** |  |  | **Without blood**  **n=42** |  |
| --- | --- | --- | --- | --- | --- | --- | --- |
|  |  | **Control lungs** | **IV bacteria** | **Airspace bacteria** | **Control lungs** | **IV bacteria** | **Airspace bacteria** |
|  |  | n=21 | n=19 | n=13 | n=17 | n=22 | n=3 |
| **IL-6** | **0h** | 9545 (3641, 14199) | 3023 (2093, 14210) | 6365 (1660, 12437) | 7203 (3284, 10675) | 8874 (3280, 22195) | 8816 (5114, 22874) |
|  | **4h** | 132746 (63735, 170308) | 118951 (68193, 145175) | 63003 (47642, 123715) | 154512 (112826, 210624) | 222067 (154782, 301572) | 192817 (117088, 464760) |
|  | **Delta** | 119862 (51705, 155378) | 108689 (65998, 138344) | 58087 (46662, 105947) | 151234 (102151, 204468) | 213124 (149948, 280306) | 169943 (108271, 459646) |
|  | **Fold change** | 10.7 (5.6, 25.1) | 28.5 (10.6, 55.3) | 12.8 (4.8, 32) | 28.5 (16.6, 44.1) | 26.9 (13.1, 65.9) | 13.3 (8.4, 90.9) |
| **IL-8** | **0h** | 6679 (2521, 10597) | 7859 (1732, 12050) | 5518 (4253, 8943) | 5587 (3901, 11270) | 6715 (3165, 21721) | 16363 (10749, 32502) |
|  | **4h** | 82824 (58046, 123804) | 128380 (58003, 168450) | 80604 (57557, 112940) | 103785 (62702, 170550) | 184847 (125646, 236966) | 147788 (80640, 421859) |
|  | **Delta** | 63652 (50754, 114201) | 120522 (56390, 132177) | 73055 (53570, 104306) | 97426 (60670, 152688) | 176878 (120836, 222870) | 115287 (69891, 405496) |
|  | **Fold change** | 11.2 (7.2, 17.7) | 16.3 (12.5, 33.8) | 13.1 (9.2, 18.6) | 14.6 (12.2, 25.7) | 27.8 (13.2, 46.9) | 7.5 (4.5, 25.8) |
| **sTNFR1** | **0h** | 1739 (1224, 2237) | 1683 (1279, 2078) | 1932 (1670, 2577) | 1868 (1632, 2585) | 2023 (1577, 2511) | 1849 (1543, 2760) |
|  | **4h** | 2912 (2249, 3390) | 2803 (2184, 4714) | 3168 (2577, 3602) | 3580 (2181, 4326) | 3658 (2923, 4460) | 4358 (2949, 5983) |
|  | **Delta** | 1066 (775, 1431) | 1274 (750, 2636) | 1147 (616, 1473) | 1136 (868, 1917) | 1539 (839, 2472) | 2509 (1405, 3223) |
|  | **Fold change** | 1.5 (1.3, 1.9) | 1.8 (1.5, 2.1) | 1.5 (1.4, 1.9) | 1.6 (1.4, 2) | 1.8 (1.4, 2.3) | 2.2 (1.9, 2.4) |
| **Ang-2** | **0h** | 1674 (1408, 2363) | 2128 (1494, 3115) | 2595 (1796, 3144) | 1865 (1337, 2317) | 2306 (1409, 3546) | 1486 (911, 5807) |
|  | **4h** | 2556 (1918, 3884) | 3657 (2224, 4863) | 3571 (2308, 4851) | 3308 (2266, 3823) | 3663 (2900, 5691) | 4442 (3839, 4497) |
|  | **Delta** | 901 (398, 1205) | 1521 (730, 2025) | 850 (630, 1743) | 1294 (1075, 1958) | 1817 (1082, 2314) | 2956 (-1968, 3586) |
|  | **Fold change** | 1.5 (1.2, 1.7) | 1.6 (1.4, 1.9) | 1.4 (1.3, 1.7) | 1.9 (1.6, 2) | 1.9 (1.5, 2.2) | 3 (0.7, 4.9) |

*Note: Data are presented as medians with interquartile range. Concentration is in pg/mL. Lungs lacking a sample from at the second time point (4h) were not included in the calculation of Delta and Fold change.

## sTable 2: Airspace biomarker kinetics stratified by experimental conditions.

|  |  |  | **With blood** |  |  | **Without blood** |  |
| --- | --- | --- | --- | --- | --- | --- | --- |
|  |  | **Control lungs** | **IV bacteria** | **Airspace bacteria** | **Control lungs** | **IV bacteria** | **Airspace bacteria** |
|  |  | n=16 | n=13 | n=10 | n=13 | n=14 | n=2 |
| **IL-6** | **0h** | 8584 (2844, 26202) | 7656 (1014, 16786) | 3508 (1347, 13553) | 4851 (2040, 14136) | 7873 (3490, 16419) | 9064 (2816, 22161) |
|  | **4h** | 144426 (60380, 321870) | 93138 (24499, 233784) | 99408 (85274, 132415) | 97447 (35704, 165436) | 287249 (92160, 349401) | 100046 (9755, 190336) |
|  | **Delta** | 120492 (50897, 297983) | 88284 (24043, 215755) | 85585 (41831, 131068) | 91702 (21261, 162189) | 284017 (89666, 327380) | 94106 (6940, 181272) |
|  | **Fold change** | 11.9 (5, 29.3) | 19.8 (13, 30.8) | 22.6 (8.4, 46.3) | 17.9 (8.7, 37.1) | 29.8 (9, 59) | 12.2 (3.5, 21) |
| **IL-8** | **0h** | 15269 (6002, 51883) | 13992 (3325, 37501) | 15163 (8150, 33755) | 16038 (7361, 33697) | 15931 (8993, 36918) | 25695 (10743, 40572) |
|  | **4h** | 171119 (129343, 294532) | 203955 (72186, 370472) | 161653 (93230, 207251) | 112950 (85191, 235859) | 254521 (162061, 395160) | 71333 (22265, 120401) |
|  | **Delta** | 158751 (105567, 239020) | 156188 (60164, 328143) | 149328 (89263, 177742) | 96657 (56032, 228499) | 247643 (155816, 381142) | 53114 (-3430, 109658) |
|  | **Fold change** | 11.7 (5, 20.5) | 11.3 (8.8, 14.2) | 8.6 (6.1, 16.9) | 7.9 (3.9, 14.8) | 18 (4.8, 28.2) | 6 (0.9, 11.2) |
| **sTNFR1** | **0h** | 1962 (1368, 3049) | 1705 (1084, 2954) | 2571 (1717, 2770) | 2068 (1599, 4721) | 2555 (1738, 3639) | 1457 (1332, 2596) |
|  | **4h** | 4236 (2406, 4742) | 5166 (2858, 6616) | 3391 (2594, 4085) | 3281 (1709, 6548) | 3874 (1940, 5759) | 2188 (750, 3626) |
|  | **Delta** | 1677 (415, 2281) | 3247 (1517, 3780) | 1196 (646, 1518) | 1217 (-30, 3454) | 909 (-184, 2119) | 162 (-1846, 2169) |
|  | **Fold change** | 1.7 (1.2, 2.5) | 2.1 (1.6, 2.7) | 1.5 (1.3, 1.6) | 1.8 (1, 2) | 1.3 (0.9, 2.4) | 1.4 (0.3, 2.5) |
| **Ang-2** | **0h** | 389 (205, 1135) | 638 (97, 1911) | 1842 (691, 2908) | 169 (106, 310) | 347 (141, 769) | 343 (202, 581) |
|  | **4h** | 1547 (1092, 2108) | 3362 (1793, 4103) | 2313 (1938, 3453) | 692 (277, 2143) | 1367 (845, 2537) | 1596 (20, 3172) |
|  | **Delta** | 615 (185, 1320) | 1493 (882, 2534) | 361 (215, 1508) | 231 (27, 690) | 776 (254, 1046) | 1205 (-182, 2591) |
|  | **Fold change** | 3.5 (1.5, 5.8) | 2.2 (1.9, 3.6) | 1.6 (1.1, 3) | 2.6 (1.3, 5.4) | 3.9 (2.2, 7.3) | 2.8 (0.1, 5.5) |

*Note: Data are presented as medians with interquartile range. Concentration is in pg/mL. Lungs lacking a sample from at the second time point (4h) were not included in the calculation of Delta and Fold change.

## sTable 3: Impact of exogenous fresh whole blood on perfusate and airspace biomarker change during 4 hours of *ex vivo* perfusion in control lungs and in lungs exposed to IV *S. pneumoniae*.

|  | **Biomarker** | **Concentration change in lungs with blood   (95% CI)** | **Difference in concentration change without blood   (95% CI)** | **P value^*^** |
| --- | --- | --- | --- | --- |
| **Control lungs** | | | | |
| **Perfusate** | **n** | 21 | 17 |  |
|  | **IL-6** | 147022 (78821, 215223) | 15364 (-62318, 93047) | 0.39 |
|  | **IL-8** | 88869 (58062, 119676) | 29778 (-16706, 76262) | 0.21 |
|  | **sTNFR1** | 878 (440, 1316) | 398 (-151, 948) | 0.16 |
|  | **Ang-2** | 990 (567, 1414) | 524 (-50, 1098) | 0.07 |
| **Airspace** | **n** | 16 | 13 |  |
|  | **IL-6** | 216148 (95734, 336561) | -73669 (-222783, 75445) | 0.33 |
|  | **IL-8** | 187736 (129999, 245474) | 33839 (-16304, 83982) | 0.58 |
|  | **sTNFR1** | 1332 (492, 2171) | 229 (-1388, 1847) | 0.78 |
|  | **Ang-2** | 934 (448, 1421) | -130 (-944, 683) | 0.75 |
| **Lungs exposed to IV *S. Pneumoniae*** | | | | |
| **Perfusate** | **n** | 19 | 22 |  |
|  | **IL-6** | 121456 (83582, 159330) | 133885 (45431, 222339) | 0.003 |
|  | **IL-8** | 122667 (82749, 162585) | 101874 (-4981, 208728) | 0.06 |
|  | **sTNFR1** | 1843 (1052, 2634) | -221 (-1115, 674) | 0.63 |
|  | **Ang-2** | 1854 (1069, 2640) | 329 (-795, 1453) | 0.57 |
| **Airspace** | **n** | 13 | 14 |  |
|  | **IL-6** | 171183 (56110, 286256) | 85258 (-68463, 238980) | 0.28 |
|  | **IL-8** | 218812 (108208, 329416) | 53807 (-103224, 210837) | 0.50 |
|  | **sTNFR1** | 2797 (1930, 3665) | -1793 (-3138, -448) | 0.009 |
|  | **Ang-2** | 1701 (1056, 2346) | -617 (-1702, 468) | 0.27 |

*P values obtained using generalized estimating equation models (GEE) using robust standard errors.

## sTable 4: Impact of cold ischemia time and baseline AFC on relationship of perfusate and airspace biomarker change in lungs perfused with compared to without blood in control and IV *S. pneumoniae* exposed lungs.

|  |  |  | **Control lungs** | | **IV *S. pneumoniae*** | |
| --- | --- | --- | --- | --- | --- | --- |
|  | **Biomarker** | **Adjustment variable** | **Concentration change    (95% CI)** | **P value^*^** | **Concentration change    (95% CI)** | **P value^*^** |
| **Perfusate** | **IL-6** | **Cold ischemia time** | -1029 (-3514, 1455) | 0.42 | -1085 (-2586, 416) | 0.16 |
|  |  | **Baseline AFC** | 982 (-557, 2522) | 0.21 | 853 (-50, 1757) | 0.06 |
|  | **IL-8** | **Cold ischemia time** | -425 (-1927, 1077) | 0.58 | -2374 (-4337, -411) | 0.02 |
|  |  | **Baseline AFC** | 594 (-274, 1461) | 0.18 | 421 (-702, 1544) | 0.46 |
|  | **sTNFR1** | **Cold ischemia time** | -6 (-29, 17) | 0.60 | -21 (-76, 34) | 0.45 |
|  |  | **Baseline AFC** | 7 (-11, 24) | 0.47 | 1 (-24, 27) | 0.91 |
|  | **Ang-2** | **Cold ischemia time** | -1 (-32, 30) | 0.96 | 11 (-63, 85) | 0.78 |
|  |  | **Baseline AFC** | 13 (-9, 36) | 0.24 | -10 (-69, 48) | 0.73 |
| **Airspace** | **IL-6** | **Cold ischemia time** | 1018 (-3344, 5380) | 0.65 | 909 (-1665, 3484) | 0.49 |
|  |  | **Baseline AFC** | 3057 (264, 5849) | 0.03 | 2933 (280, 5586) | 0.03 |
|  | **IL-8** | **Cold ischemia time** | 1541 (-566, 3649) | 0.15 | -906 (-3961, 2149) | 0.56 |
|  |  | **Baseline AFC** | 1747 (473, 3021) | 0.01 | 3280 (602, 5958) | 0.02 |
|  | **sTNFR1** | **Cold ischemia time** | -12 (-90, 66) | 0.77 | 0 (-62, 61) | 0.99 |
|  |  | **Baseline AFC** | 46 (11, 82) | 0.01 | -6 (-41, 30) | 0.76 |
|  | **Ang-2** | **Cold ischemia time** | -13 (-35, 10) | 0.27 | 25 (-16, 67) | 0.23 |
|  |  | **Baseline AFC** | -1 (-20, 18) | 0.92 | -21 (-58, 16) | 0.27 |

*P values obtained using generalized estimating equation models (GEE) using robust standard errors with interaction terms for cold ischemia time or baseline AFC.

## sTable 5: Impact of bacteria on perfusate and airspace biomarker change during 4 hours of *ex vivo* perfusion in control lungs relative to lungs exposed to IV *S. pneumoniae* with blood, IV *S. pneumoniae* without blood, or airspace *S. pneumoniae* with blood.

|  | **Biomarker** | **Concentration change in control lungs   (95% CI)** | **Difference in concentration change with bacteria (95% CI)** | **P value^*^** |
| --- | --- | --- | --- | --- |
| **IV *S. pneumoniae* with exogenous blood** | | | | |
| **Perfusate** | **n** | 21 | 19 |  |
|  | **IL-6** | 147030 (78843, 215217) | -25574 (-103579, 52431) | 0.52 |
|  | **IL-8** | 88844 (58066, 119622) | 33823 (-16592, 84238) | 0.19 |
|  | **sTNFR1** | 876 (438, 1314) | 967 (62, 1872) | 0.04 |
|  | **Ang-2** | 990 (567, 1414) | 864 (-28, 1757) | 0.06 |
| **Airspace** | **n** | 16 | 13 |  |
|  | **IL-6** | 216385 (95700, 337069) | -45512 (-211978, 120954) | 0.59 |
|  | **IL-8** | 187610 (129984, 245235) | 30819 (-93566, 155203) | 0.63 |
|  | **sTNFR1** | 1318 (466, 2170) | 1492 (273, 2711) | 0.02 |
|  | **Ang-2** | 915 (431, 1400) | 823 (8, 1639) | 0.05 |
| **IV *S. pneumoniae* without exogenous blood** | | | | |
| **Perfusate** | **n** | 17 | 22 |  |
|  | **IL-6** | 162600 (126063, 199138) | 92741 (4830, 180653) | 0.04 |
|  | **IL-8** | 120611 (85361, 155862) | 103915 (-1316, 209146) | 0.05 |
|  | **sTNFR1** | 1284 (955, 1613) | 338 (-193, 869) | 0.21 |
|  | **Ang-2** | 1514 (1126, 1902) | 671 (-222, 1563) | 0.14 |
| **Airspace** | **n** | 13 | 14 |  |
|  | **IL-6** | 142415 (56441, 228389) | 114710 (-19905, 249324) | 0.10 |
|  | **IL-8** | 159922 (79321, 240523) | 112587 (-24578, 249751) | 0.11 |
|  | **sTNFR1** | 1590 (215, 2964) | -582 (-2290, 1126) | 0.50 |
|  | **Ang-2** | 772 (99, 1445) | 309 (-793, 1411) | 0.58 |
| **Airspace *S. pneumoniae* with exogenous blood** | | | | |
| **Perfusate** | **n** | 21 | 13 |  |
|  | **IL-6** | 147005 (78693, 215317) | -70135 (-143563, 3294) | 0.06 |
|  | **IL-8** | 88830 (57997, 119664) | -2249 (-45635, 41137) | 0.92 |
|  | **sTNFR1** | 877 (438, 1316) | 163 (-393, 719) | 0.57 |
|  | **Ang-2** | 987 (563, 1410) | 266 (-344, 877) | 0.39 |
| **Airspace** | **n** | 16 | 10 |  |
|  | **IL-6** | 215494 (95379, 335609) | -109136 (-236958, 18686) | 0.09 |
|  | **IL-8** | 187418 (129947, 244888) | -49560 (-117608, 18487) | 0.15 |
|  | **sTNFR1** | 1325 (478, 2172) | -184 (-1112, 744) | 0.70 |
|  | **Ang-2** | N/A | N/A | N/A |

*P values obtained using generalized estimating equation models (GEE) using robust standard errors.

## sTable 6: Impact of cold ischemia time, baseline AFC, and pre-procurement antibiotics on relationship of perfusate and airspace biomarker change in control compared to IV *S. pneumoniae* exposed lungs perfused with and without blood.

|  |  |  | **With blood** | | **Without blood** | |
| --- | --- | --- | --- | --- | --- | --- |
|  | **Biomarker** | **Adjustment variable** | **Concentration change    (95% CI)** | **P value^*^** | **Concentration change    (95% CI)** | **P value^*^** |
| **Perfusate** | **IL-6** | **Cold ischemia time** | -1114 (-3195, 967) | 0.29 | -970 (-2759, 819) | 0.29 |
|  |  | **Baseline AFC** | 1100 (-141, 2340) | 0.08 | 575 (-575, 1725) | 0.33 |
|  |  | **Pre-procurement antibiotics** | -9553 (-72937, 53831) | 0.77 | -32498 (-90501, 25505) | 0.27 |
|  | **IL-8** | **Cold ischemia time** | -1371 (-2827, 85) | 0.07 | -1443 (-3699, 814) | 0.21 |
|  |  | **Baseline AFC** | 657 (-144, 1458) | 0.11 | 227 (-1148, 1603) | 0.75 |
|  |  | **Pre-procurement antibiotics** | -1358 (-39550, 36834) | 0.94 | -16598 (-89205, 56009) | 0.65 |
|  | **sTNFR1** | **Cold ischemia time** | -23 (-66, 19) | 0.28 | 4 (-26, 34) | 0.80 |
|  |  | **Baseline AFC** | -4 (-24, 15) | 0.65 | 20 (1, 39) | 0.04 |
|  |  | **Pre-procurement antibiotics** | -280 (-1081, 521) | 0.49 | -1003 (-1653, -353) | 0.002 |
|  | **Ang-2** | **Cold ischemia time** | 10 (-46, 66) | 0.72 | -4 (-52, 43) | 0.86 |
|  |  | **Baseline AFC** | -5 (-36, 26) | 0.77 | 14 (-49, 77) | 0.66 |
|  |  | **Pre-procurement antibiotics** | 150 (-950, 1249) | 0.79 | -1593 (-3361, 174) | 0.08 |
| **Airspace** | **IL-6** | **Cold ischemia time** | 799 (-2948, 4546) | 0.68 | 1319 (-1576, 4213) | 0.37 |
|  |  | **Baseline AFC** | 4129 (1671, 6588) | 0.001 | 1023 (-1573, 3619) | 0.44 |
|  |  | **Pre-procurement antibiotics** | 52662 (-44853, 150176) | 0.29 | -30953 (-111978, 50073) | 0.45 |
|  | **IL-8** | **Cold ischemia time** | 480 (-1673, 2632) | 0.66 | 100 (-3600, 3800) | 0.96 |
|  |  | **Baseline AFC** | 3099 (1532, 4667) | <0.0001 | 1536 (-1159, 4231) | 0.26 |
|  |  | **Pre-procurement antibiotics** | 38558 (-18093, 95209) | 0.18 | 4008 (-72793, 80809) | 0.92 |
|  | **sTNFR1** | **Cold ischemia time** | -51 (-102, 0) | 0.05 | 69 (-25, 163) | 0.15 |
|  |  | **Baseline AFC** | 23 (-7, 53) | 0.13 | 17 (-30, 63) | 0.48 |
|  |  | **Pre-procurement antibiotics** | 506 (-830, 1841) | 0.46 | -1601 (-3268, 66) | 0.06 |
|  | **Ang-2** | **Cold ischemia time** | -1 (-29, 28) | 0.96 | 16 (-23, 56) | 0.42 |
|  |  | **Baseline AFC** | -4 (-23, 16) | 0.71 | -23 (-69, 23) | 0.32 |
|  |  | **Pre-procurement antibiotics** | 392 (-396, 1180) | 0.33 | -1687 (-3395, 22) | 0.05 |

*P values obtained using generalized estimating equation models (GEE) using robust standard errors with interaction terms for cold ischemia time, baseline AFC, or administration of antibiotics prior to organ procurement.

## sTable 7: Impact of cold ischemia time, baseline alveolar fluid clearance, and pre-procurement antibiotics on relationship of perfusate and airspace biomarker change in control compared to airspace *S. pneumoniae* exposed lungs perfused with blood.

|  | **Biomarker** | **Adjustment variable** | **Concentration change    (95% CI)** | **P value^*^** |
| --- | --- | --- | --- | --- |
| **Perfusate** | **IL-6** | **Cold ischemia time** | -1157 (-3344, 1030) | 0.30 |
|  |  | **Baseline AFC** | 859 (-692, 2410) | 0.28 |
|  |  | **Pre-procurement antibiotics** | -30504 (-123272, 62264) | 0.52 |
|  | **IL-8** | **Cold ischemia time** | -641 (-1836, 555) | 0.29 |
|  |  | **Baseline AFC** | 430 (-414, 1273) | 0.32 |
|  |  | **Pre-procurement antibiotics** | -13048 (-62325, 36229) | 0.60 |
|  | **sTNFR1** | **Cold ischemia time** | 6 (-14, 26) | 0.55 |
|  |  | **Baseline AFC** | -4 (-21, 13) | 0.67 |
|  |  | **Pre-procurement antibiotics** | -165 (-858, 529) | 0.64 |
|  | **Ang-2** | **Cold ischemia time** | 15 (-17, 47) | 0.35 |
|  |  | **Baseline AFC** | -5 (-28, 17) | 0.64 |
|  |  | **Pre-procurement antibiotics** | -535 (-1647, 577) | 0.35 |
| **Airspace** | **IL-6** | **Cold ischemia time** | 312 (-3617, 4240) | 0.88 |
|  |  | **Baseline AFC** | 3295 (464, 6127) | 0.02 |
|  |  | **Pre-procurement antibiotics** | 22963 (-113740, 159666) | 0.74 |
|  | **IL-8** | **Cold ischemia time** | 703 (-1277, 2684) | 0.49 |
|  |  | **Baseline AFC** | 1975 (760, 3190) | 0.001 |
|  |  | **Pre-procurement antibiotics** | 6665 (-53989, 67319) | 0.83 |
|  | **sTNFR1** | **Cold ischemia time** | -28 (-69, 13) | 0.19 |
|  |  | **Baseline AFC** | 24 (-7, 55) | 0.13 |
|  |  | **Pre-procurement antibiotics** | 70 (-1603, 1744) | 0.93 |
|  | **Ang-2** | **Cold ischemia time** | N/A | N/A |
|  |  | **Baseline AFC** | N/A | N/A |
|  |  | **Pre-procurement antibiotics** | N/A | N/A |

*P values obtained using generalized estimating equation models (GEE) using robust standard errors with interaction terms for cold ischemia time, baseline AFC, or administration of antibiotics prior to organ procurement.
